# Supplementary material for: Extracorporeal Removal of Thermosensitive Liposomal Doxorubicin from Systemic Circulation after Tumor Delivery to Reduce Toxicities
Source: Cancers (Basel). 2022 Mar 4;14(5):1322. doi: 10.3390/cancers14051322 (PMC8909191; doi:10.3390/cancers14051322)
Supplement: Supplementary file 1 [file cancers-14-01322-s001.zip › cancers-1613488-supplementary.pdf]

Supplementary Materials

TSL-based delivery only occurs during Hyperthermia

Several prior studies have demonstrated that for the newer TSL formulations which are based on intravascular triggered delivery [1–3], drug delivery to cancer cells only occurs while hyperthermia is applied (Figure S1) [1–4].

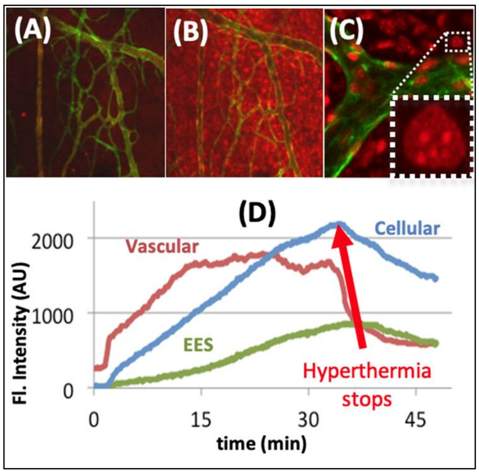

Figure S1. Intravital TSL-Dox study, where a tumor was imaged *in vivo* during hyperthermia: Endothelial cells (green) and Dox (red). Images show Dox tissue accumulation and cell uptake: **(A)** 5 min, **(B)** 20 min following hyperthermia-induced release from TSL (FOV 500x500 μm). **(C)** Subcellular Dox localization (FOV 150x150 μm). **(D)** Aggregate fluorescence within FOV of intravascular, interstitial (EES), and cellular regions from data in (**A**,**B**). Cell uptake discontinues once hyperthermia stops (red arrow). Figure reproduced with permission from [5].

Filter efficacy drops during in vivo filtration

While initially filtration efficacy was high (~80%), a significant degradation was observed (Figure S2) suggesting further improvements in performance of the proposed method is possible with advanced filter designs.

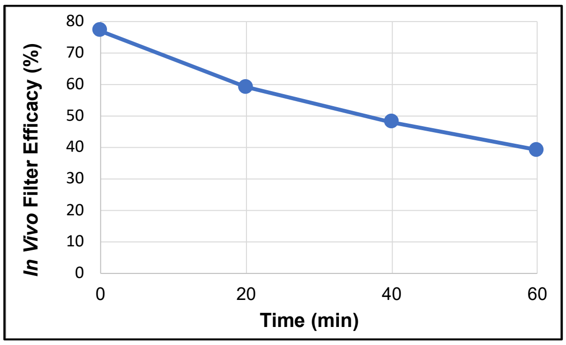

Figure S2. *In vivo* filter efficacy drops from ~80% to ~40% during 60 min extracorporeal circuit filtration.

Fluorescence monitoring of drug removal during filtration

We performed an initial *in vitro* study where TSL-Dox in plasma was pumped through an imaging module, before heating, after heating (42 °C), and after filtration (Figure S3).

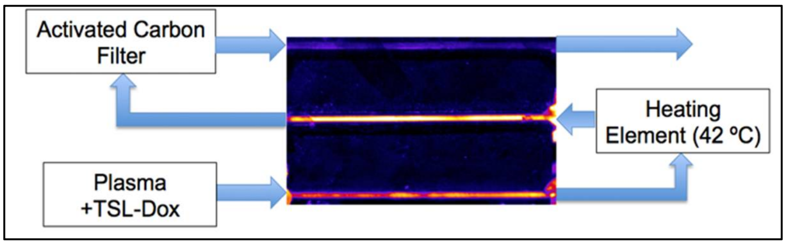

Figure S3. In vitro study to demonstrate fluorescence monitoring of drug filtration. For initial in vitro studies, we built an module with three tubes (shown in center) that allows real-time fluorescence imaging during filtration. This fluorescence image shows encapsulated drug with plasma entering the bottom tube. Fluorescence increases after heat-induced drug release from TSL (middle tube). Plasma with released drug was then passed through a carbon filter. Marginal fluorescence after filtration demonstrates filter efficacy (top tube). In in vivo studies, similar imaging was performed in blood rather than plasma.

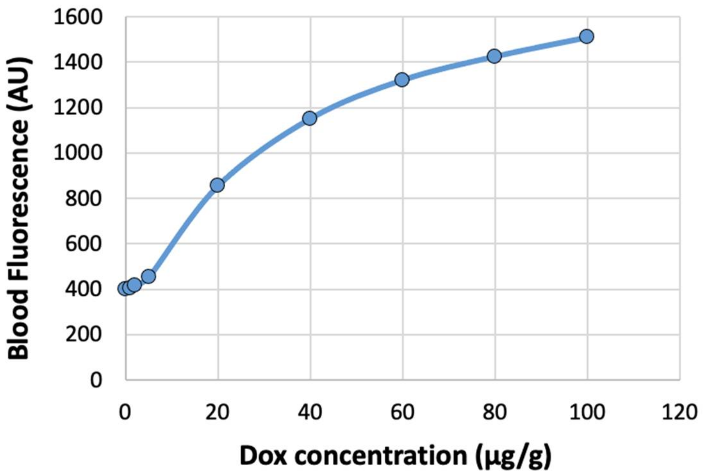

Figure S4. Standard curve of blood fluorescence vs. Dox concentration. Blood was doped with varying amounts of Dox, and fluorescence was measured in the capillary tubes of the imaging module. While there was significant background fluorescence present, Dox concentration could be determined with reasonable accuracy.

Computer model

The computer model was adapted from a prior publication [3], with two changes: (1) the physiological model parameters were adapted to rat physiology, as the prior publication considered mouse physiology; and (2) the extracorporeal circuit with filtration was integrated (Figure 9). We did however not implement any delay in the ECC, i.e. blood after ECC filtration immediately returns to systemic circulation.

Table S1. Complete list of model parameters. Parameter variable names are noted in brackets.

Model Parameter	Parameter Value	Comments
Animal weight	250 g	From in vivo experiments
Systemic blood volume ($V_b{}^B$)	16 mL	Estimated from [6]
Hematocrit (*Hct*)	0.45	
Systemic plasma volume ($V_p{}^B$)	8.8 mL	$V_p{}^B = V_b{}^B$ *(1-*Hct*)
Dosage	7 mg/kg (=1.75 mg)	Bolus injection was assumed
Volume of ECC (V_{ECC})	4.2 mL	From in *vivo* experiments
Filter perfusion rate (F_{filt})	0.35 ml/min	Filtration started 30 min after injection
Filtration Efficacy (Eff_{filt})	55%, 80%, 100%	
Half-life of TSL-Dox	55 min	Experimentally determined
Volume of distribution of unencapsulated Dox (V_D)	237.5 mL	Scaled from mice to rates based on prior study [7]
Dox transfer rate constant plasma->systemic tissue (k_p)	9.4e-3 s^{-1}	Calculated from prior study [7]
Dox transfer rate constant systemic tissue->plasma (k_t)	7.05e-5 s^{-1}	Calculated from prior study [7]
Dox clearance rate constant (k_e)	2.1e-3 s^{-1}	Calculated from prior study [7]
Dox transfer rate constant plasma->cardiac tissue (k_{ph})	8.3e-3 s^{-1}	Calculated from prior study [7]
Dox transfer rate constant plasma->cardiac tissue (k_{hp})	1.15e-4 s^{-1}	Calculated from prior study [7]
TSL-Dox clearance rate constant (k_{e_TSL})	4.38e-5 s^{-1}	Calculated from prior study [8]
TSL-Dox leakage rate at body temperature (R_{37})	9.17e-5 s^{-1}	Calculated from prior study [8]

Computer model equations

Systemic plasma concentration of TSL-encapsulated Dox:

$$\frac{dc_{p\_Lip}}{dt} = -c_{p\_Lip} \cdot R_{37} - c_{p\_Lip} \cdot k_{e\_LTSL} - c_{p\_Lip} \cdot \frac{F_{filt}}{V_p{}^B} \qquad \text{(Equation S1)}$$

Systemic plasma concentration of unencapsulated Dox:

$$\frac{dc_p{}^B}{dt} = c_{p_{Lip}} \cdot F \quad R_{37} \cdot \frac{V_p{}^B}{V_D} + c_{p_{Lip}} \cdot \frac{F_{filt}}{V_D} \cdot (1 - Eff_{filt}) \qquad \text{(Equation S2)}$$

Dox concentration accumulated in ECC filter:

$$\frac{dc_{ECC}}{dt} = c_{p_{Lip}} \cdot \frac{F_{filt}}{V_{ECC}} \cdot Eff_{filt} \quad \text{(Equation S3)}$$

Systemic tissue concentration of Dox:

$$\frac{dc_t{}^B}{dt} = k_p c_p{}^B - k_t c_t{}^B \quad \text{(Equation S4)}$$

Cardiac tissue concentration of Dox:

$$\frac{dc_t{}^H}{dt} = k_{ph} c_p{}^B - k_{hp} c_t{}^H \quad \text{(Equation S5)}$$

As in a prior publication, these ordinary differential equations were solved in the software Matlab v2020a [3].

References

1. Manzoor, A.A.; Lindner, L.H.; Landon, C.D.; Park, J.Y.; Simnick, A.J.; Dreher, M.R.; Das, S.; Hanna, G.; Park, W.; Chilkoti, A.; et al. Overcoming limitations in nanoparticle drug delivery: Triggered, intravascular release to improve drug penetration into tumors. *Cancer Res.* **2012**, *72*, 5566–5575.
2. Li, L.; Ten Hagen, T.L.; Hossann, M.; Suss, R.; van Rhoon, G.C.; Eggermont, A.M.; Haemmerich, D.; Koning, G.A. Mild hyperthermia triggered doxorubicin release from optimized stealth thermosensitive liposomes improves intratumoral drug delivery and efficacy. *J. Control. Release Off. J. Control. Release Soc.* **2013**, *168*, 142–150.
3. Gasselhuber, A.; Dreher, M.R.; Rattay, F.; Wood, B.J.; Haemmerich, D. Comparison of conventional chemotherapy, stealth liposomes and temperature-sensitive liposomes in a mathematical model. *PLoS ONE* **2012**, *7*, e47453.
4. Motamarry, A.; Negussie, A.H.; Rossmann, C.; Small, J.; Wolfe, A.M.; Wood, B.J.; Haemmerich, D. Real-time fluorescence imaging for visualization and drug uptake prediction during drug delivery by thermosensitive liposomes. *Int. J. Hyperth. Off. J. Eur. Soc. Hyperthermic Oncol. N. Am. Hyperth. Group* **2019**, *36*, 817–826.
5. Motamarry, A.; Haemmerich, D. Thermosensitive liposomes. In *Liposomes*; Catala, A., Ed. IntechOpen: Rijeka, Croatia, 2017; pp. 187–211.
6. Lee, H.B.; Blaufox, M.D. Blood volume in the rat. *J. Nucl. Med. Off. Publ. Soc. Nucl. Med.* **1985**, *26*, 72–76.
7. van der Vijgh, W.J.F.; Maessen, P.A.; Pinedo, H.M. Comparative metabolism and pharmacokinetics of doxorubicin and 4′-epidoxorubicin in plasma, heart and tumor of tumor-bearing mice. *Cancer Chemother. Pharmacol.* **1990**, *26*, 9–12.
8. Lokerse, W.J.; Kneepkens, E.C.; Ten Hagen, T.L.; Eggermont, A.M.; Grull, H.; Koning, G.A. In depth study on thermosensitive liposomes: Optimizing formulations for tumor specific therapy and in vitro to in vivo relations. *Biomaterials* **2016**, *82*, 138–150.
